# A participatory intervention to improve the mental health of widows of injecting drug users in north-east India as a strategy for HIV prevention

**DOI:** 10.1186/1472-698X-7-3

**Published:** 2007-04-19

**Authors:** Alexandra Devine, Michelle Kermode, Prabha Chandra, Helen Herrman

**Affiliations:** 1 Australian International Health Institute, Alan Gilbert Building, Level 5 Barry St, University of Melbourne, Carlton, 3010, Victoria, Australia; 2 Australian International Health Alan Gilbert Building, Level 5 Barry St, University of Melbourne, Carlton, 3010, Victoria, Australia; 3 Dept. of Psychiatry, National Institute of Mental Health & Neurosciences, Hosur Road Bangalore – 560029, India; 4 Australian International Health Alan Gilbert Building, Level 5 Barry St, University of Melbourne, Carlton, 3010, Victoria, Australia

## Abstract

**Background:**

Manipur and Nagaland, in the north-east of India, are classified as high prevalence states for HIV, and intravenous drug use is an important route of transmission. Most injecting drug users (IDUs) are men, an estimated 40% are married, and death rates have been high in the last five years, consequently the number of widows of IDUs has increased. Many of these widows and their children are HIV-infected and experience poor health, discrimination, and impoverishment; all factors likely to be compromising their mental health. People with poor mental health are more likely to engage in HIV risk behaviours. Mental health can be promoted by public health actions with vulnerable population groups.

**Methods:**

We designed an intervention study to assess the feasibility and impact of a participatory action process to promote the mental health and well-being of widows of IDUs in Manipur and Nagaland, as a strategy for reducing the risk of engagement in HIV risk behaviours. This paper describes the background and rationale for the study, the intervention, and the study methods in detail.

**Results:**

Pending analysis.

**Conclusion:**

This intervention study will make a significant contribution to the emerging evidence that supports associations between mental health and HIV. The concept of promoting mental health among women who are vulnerable to HIV infection or already infected as a strategy for HIV prevention in a development setting is breaking new ground.

## Background

### HIV/AIDS in north-east India

Two of the north-east states of India, Manipur and Nagaland, are classified as high prevalence states for HIV (HIV prevalence in antenatal women >1%) [1]. Intravenous drug use (IDU) in these states is an important route of HIV transmission [2]. A constellation of social factors including political instability, unemployment, and easy availability of heroin from across the Myanmar border and cheap narcotic-based pharmaceutical agents, all contribute to a high prevalence of injecting drug use in this part of the world, especially among young men [3,4]. Recent reports estimate that injecting drug users (IDUs) constitute 1.9–2.7% of the adult population [2]. In 2005, the HIV prevalence among IDUs in Manipur and Nagaland was estimated to be 24% and 5% respectively, representing an increase in both states from the previous year (NACO 2006). In a sample of IDUs in the north-east, 75% were found to be HIV positive [2].

Most IDUs are men, an estimated 40% are married [5], and death rates have been high in the last five years; consequently the number of widows of IDUs has increased [4]. HIV transmission from IDUs to their sexual partners and wives has been documented [2,6]. Women in India are often socially and economically disadvantaged following the death of their husband. For widows of injecting drug users (IDUs) the situation is arguably worse due to their increased risk of infection with HIV and other blood-borne viruses, and the increased likelihood of stigma and discrimination.

A situation assessment conducted among widows of IDUs in Manipur during 2004 found that many were faced with a range of socio-economic, health and psychosocial problems, including poverty, grief, loneliness, discrimination, illness associated with HIV infection, difficulty providing care and support for their children (some of whom are also living with HIV); all factors likely to be compromising their mental health [7]. Some widows reported engaging in HIV risk behaviours including alcohol and substance misuse, sex work and unprotected sex. Accessing HIV prevention services was not a priority for these women who were predominantly concerned about livelihood issues and their children's well-being [7]. Additionally, insurgent groups in both states target IDUs and sex workers with public humiliation and violence, driving these populations underground and making it difficult to reach them with HIV prevention programs.

With this situation assessment in mind, we designed an intervention study that uses a participatory action process to promote the mental health and well-being of widows of IDUs in Manipur and Nagaland, as a strategy for reducing engagement in HIV risk behaviours. This paper outlines the background and rationale for the intervention, and then describes the study design in detail.

### Links between mental health and HIV/AIDS

A growing body of evidence links mental health with HIV/AIDS in a range of ways. People with poor mental health, including those with untreated mental illnesses such as depression tend to have impaired judgement, impulsive behaviour, reduced fear of consequences, and increased vulnerability to outside influences, and as a result are more likely to engage in HIV risk behaviours [3,8]. Interactions between drug and alcohol use and depression are common, often leading to a decreased concern for personal safety. This has important implications for HIV prevention but limited relevant research has been conducted in India and elsewhere [3,8-10].

People living with HIV/AIDS have an increased risk of developing mental health problems including depression and substance misuse [3,9]. These conditions adversely affect HIV/AIDS treatment adherence, contribute to risk behaviours and exacerbate social difficulties associated with stigma and discrimination. In itself, depression is at present the greatest overall source of disability in the world [8]. Integrating mental health interventions with HIV/AIDS care has the potential to benefit both the mental and physical health of people living with HIV/AIDS [3,8-10].

### Mental health promotion

Mental health is described by WHO as an integral component of health, and as: 'a state of well-being in which the individual realises his or her own abilities, can cope with the normal stresses of life, can work productively and fruitfully, and is able to make a contribution to his or her community' [11]. Mental health is not simply the absence of mental illness. It is the foundation for well-being and effective functioning of individuals and communities [12]. Poor mental health predisposes people to mental illnesses, which are common in all populations. Mental illnesses are associated in all settings with indicators of poverty; including low levels of education, poor housing and low income [13], and with other illnesses including HIV infection [9]. Substance misuse, violence and health problems such as HIV and depression are more prevalent and more difficult to cope with in conditions of low income, limited education and unemployment [14].

Emerging evidence indicates that mental health can be promoted by public health actions with vulnerable population groups [12]. Just as physical health can be promoted, so too can mental health. A recent WHO report [12] draws on a public health framework proposed initially by the Victorian Health Promotion Foundation [15,16] that identifies three key social and economic determinants of community and individual mental health: social inclusion, freedom from discrimination and violence, and access to economic resources.

Social inclusion is characterised by strong social relationships and networks, involvement in community activities and civic engagement. Social exclusion refers to the process whereby an individual or group is disconnected from the socio-economic, political and cultural system of their community. People who experience social exclusion are more at risk of poor mental and physical health [15].

Discrimination is the process by which members of a socially defined group are treated differently (generally unfairly) because of their membership of that group. People may experience many forms of discrimination based on race, ethnicity, sex, age and religion. Discrimination is associated with reduced well-being, low self esteem and major depression [15]

Access to economic resources refers not only to employment but also to factors that promote employment such as education. It also includes being able to feed, clothe and house oneself and one's family. People without sufficient access to economic resources are at higher risk of poor mental health [15].

Mental health promotion aims to achieve better mental health and wellbeing by improving the social, physical and economic environments that influence mental health [15]. Psychosocial and environmental factors influence (protect or negate) a number of health behaviours. Actions to promote mental health can be designed to foster protective factors in individuals such as coping capacity and resilience; increase connections between individuals and communities; create opportunities for income generation and employment; assist in community mobilisation; and address stigma and discrimination [15].

### Participatory interventions in health development

While evidence exists that behaviour change communication interventions contribute to a reduction in HIV risk behaviours, it is also clear that high risk behaviours continue to increase in some parts of the world [17]. Interventions designed to reduce HIV risk need to extend to the broader range of mental health promotion actions noted above, and include community participation. While the health benefits of community participation are well understood in development work, health policy does not always reflect this, partly because the evidence related to this approach is limited.

Our intervention study draws on participatory action research (PAR) approaches to health development. PAR seeks to empower the target community to actively participate in research and development activities, to identify problems and develop solutions in relation to particular research questions. This enhances self-confidence and leadership skills, and assists communities to address their own health and social needs [18-20].

A recent study has demonstrated that community based participatory action can have a significant positive effect on health outcomes, such as maternal and infant survival and morbidity [21]. The Mother and Infant Research Activities (MIRA) Makwanpur trial in Nepal is a leading example of the use of participatory interventions in health development and begins to fill a gap in the evidence base for effectiveness regarding the use of such interventions [22,23]. This trial was a cluster-randomised controlled trial of a community-based participatory intervention to reduce peri-natal and neonatal mortality rates in rural Nepal. The trial aimed to build on community planning and decision making to improve maternal and newborn care through the development of 111 women's groups. These groups were randomised into 42 clusters, and half were involved with the participatory intervention; both the control and intervention groups received improvements in health care services [23].

The intervention involved training local women to facilitate monthly meetings through a participatory action cycle of problem identification, community planning, implementation and evaluation of strategies to address the identified problems. Of the 111 women's groups, 77 went on to develop and implement strategies to address peri-natal health problems and 100 groups continued to meet to discuss peri-natal health. The trial resulted in a reduction in neonatal mortality by 30% in the intervention cluster. It also resulted in a significant decline in maternal mortality [23].

Community based participatory research is neglected in many health fields, including mental health. Similarly, the likely connections between community participation, social cohesion and the mental health of vulnerable populations, and the impact of these on HIV risk behaviours are poorly researched and merit attention.

### Appreciative Inquiry

Appreciative Inquiry (AI) is an approach to development that highlights local community strengths (relationships and assets), achievements and visions, rather than the more conventional focus on community problems, deficiencies and needs. AI assists communities to design and apply their own strategies to promote positive and sustainable change [24-26].

The AI cycle moves through four phases; Discovery, Dreaming, Designing, and Delivery/Destiny. Information about the health issue and local attributes and successes are used by the community to create a vision of what they can achieve if their strengths are mobilised. The community develops and applies strategies to address the health problem [24-26]. The AI approach underpins the intervention outlined in the following section.

We hypothesised that the development of structured and facilitated participatory action groups (PAGs) among widows of IDUs, with a focus on promoting mental health and well-being and informed by a strengths-based approach, will be associated with: (1) improved mental health; and (2) a reduced likelihood of engagement in HIV risk behaviours. We designed the following intervention study to explore this possibility.

## Methods

### Objectives

The objectives of the study are to:

1. Learn about the women's perspectives on mental health and well-being and the links between mental health and HIV

2. Assess changes in the women's quality of life and well-being during the course of the PAG meetings

3. Assess changes in engagement in HIV risk behaviours

4. Describe the process and outcome of the PAGs from the perspective of the women

5. Document the process of establishing and conducting the PAGs so it can be repeated or adapted in the future.

Widows of IDUs are defined for the purpose of the study as women whose husband or partner has died from a HIV/AIDS-related illness or from an IDU-related condition (such as overdose).

### Ethical approval

Ethics approval for the study was obtained from the University of Melbourne Human Research Ethics Committee and the Emmanuel Hospital Association (EHA) Institutional Review Board in early 2006. The study is funded by the United Kingdom's Department for International Development (DFID) through the Research and Learning Fund. A psychiatrist was identified in each state to provide a referral point for any participant experiencing a serious mental health problem.

### Research and local partnerships

Organisational partnerships have been formed between: (1); The Australian International Health Institute (AIHI) from the University of Melbourne; (2); Project ORCHID, which is collaboration between AIHI and a large Indian non-government organisation (NGO), the Emmanuel Hospital Association (EHA), which aims to increase the HIV prevention capacity of a network of NGOs in Manipur and Nagaland, and is funded by the Bill and Melinda Gates Foundation; and (3), six local NGOs, three from each state.

The inception phase of the study involved identification of three local partner NGOs in each state. Partner NGOs were consulted regarding the purpose, methods and feasibility of the study. By establishing partnerships with local NGOs the study hopes to enhance their knowledge and understanding of mental health and their capacity to work in the area, as well as increase their focus and service delivery for widows of IDUs.

Two state-based research teams were formed, one for each state. Each research team consisted of one research officer, and three NGO liaison workers (one nominated representative from each NGO). Two widows from each of the six PAGs will take the role of peer facilitator. The research officer will oversee the PAG process, provide guidance and support to the liaison workers and facilitators, collect and translate data and contribute to data analysis. The liaison workers will provide support to the facilitators, assist in documentation of the process and ensure that partner NGOs are engaged.

Each NGO brought together a group of widows for an information session during which the women were told about the intervention and the study and invited to participate. Three PAGs have been established in each state with 10–15 widows in each group.

### Training

The next phase of the study involved training the research teams. The objectives of the training were to provide an understanding of the background and rationale for the intervention and the study and to prepare the team members for the intervention and accompanying data collection. Training for the research officers and NGO liaison workers included; an overview of research, ethics, qualitative and quantitative research methods, mental health promotion, the links between mental health and HIV, PAR and AI. Training was participatory and included techniques and exercises that could also be utilised during facilitation of the PAG meetings. Following this, the research officers and NGO liaison workers actively assisted with the training of the peer facilitators. The training emphasised the importance of ensuring that the PAG process is a positive experience for facilitators and participants, and facilitators were trained in activities to help the groups identify strengths and to promote enjoyment of the sessions.

### The intervention

The intervention consists of ten peer facilitated PAG meetings for widows of IDUs. The research officers and NGO liaison workers will attend the meetings when data collection occurs and as requested by the participants and peer facilitators. The PAGs will be held every fortnight for half a day over a twenty week period. An outline of the PAG process is summarised in Table 1. The women's travel and childcare costs will be covered and refreshments provided at each session. The meeting venues, which are a secure and comfortable area for the women to meet, are being provided by the partner NGOs ensure. All meetings will be participatory, strengths-based and comprise a combination of structured activities and open discussion, with a focus on the key factors identified in the framework for mental health promotion (social inclusion, freedom from discrimination and access to economic resources). Written guidelines for each session have been developed and will be adapted by the local teams to suit the needs of each group. It is hoped that the IDU widows will ultimately own and lead the process, and that it will enhance their awareness of mental health as an important aspect of health, as well as increase their capacity to take action to improve their own lives. In the later stages of the intervention each group will participate in an action planning process to develop strategies for promoting mental health and the sustainability of the groups.

**Table 1:** Outline of the ten Participatory Action Group meetings for widows of IDUs

**Session**	**Outline**	**Expected Outcome**	**Data Collection**
**1**	**Introduction to the PAG process** • Introductory activity • Developing ground rules • Identifying members' expectations • Activity to highlight individual strengths and skills • Administering baseline questionnaires	• Promote understanding of the purpose of the intervention • Establish ground rules and expectations • Highlight focus on value of group process and strength based approach • Identify participants' skills and strengths and how these skills might be used to develop strategies to address future challenges.	• Baseline questionnaires: - WHO QOL-BREF - GHQ12 - Health Risk Questionnaire • Meeting summary report
**2**	**Concepts and determinants of mental health** • Conducting FGD 1: Concepts and determinants of mental health for widows of IDUs • Naming the group • Finishing with a fun activity	• Learn about the participants perspectives on mental health and well-being • Enhance group cohesion • End session with sense of enjoyment	• FGD transcripts • Meeting summary report
**3**	**What is mental health?** • Energising activity • Conducting a facilitator-led group discussion on mental health and illness • Mapping exercise – how to promote mental health of widows of IDUs • Free discussion time	• Develop awareness of mental health and illness • Create understanding of factors that promote mental health	• Meeting summary report
**4**	**Positive futures and support from family, friends and community – a focus on social inclusion** • Energizing activity • Envisioning a positive future activity • Brain-storming activity on promoting support from family and friends • Free discussion time	• Participants to identify factors that contribute to a positive future for individuals and the group • Introduce concept of social inclusion • Develop understanding of connection between social inclusion and mental health • Develop ideas to promote support from family, friends and community	• Meeting summary report
**5**	**A focus on stigma and discrimination** • Energizing activity • Brain-storming activity on stigma and discrimination • Teaching relaxation techniques	• Identify common sources of stigma and discrimination • Develop ideas to address stigma and discrimination • Develop understanding of the connection between stigma and discrimination and mental health • Participants to gain skills in coping with stressful situations and techniques to relax	• Meeting summary report
**6**	**A focus on access to economic resources** • Energizing activity • Conducting small group activities: reading 2 stories based on women and income generation. Discuss lessons learnt and develop group and individual ideas for income generation • Activity to identify group priority areas for income generation • Additional information provided for research officers and facilitators on income generation • Free discussion time	• Identify relationship between access to economic resources and mental health • Develop understanding of and ideas for income generation for groups and individuals • Encourage solutions that are not dependent on external funding • Identify group priority areas for income generation for further development next sessions	• Meeting summary report
**7**	**Development of action plans** • Conducting a discussion about the nature and benefits of the action planning process for groups and individuals • Develop vision and mission statements for action plan • Discussion of strategies for achieving action plans	• Create understanding of benefits of action plan • Develop group ideas on how to promote mental health • Develop capacity of groups to take action to improve their own mental health • Encourage sustainability of group process	• Meeting summary report
**8**	**Most Significant Change and action plan development** • Collection of stories for MSC • Further development of action plans • Free discussion time	• Learn about the impact of the intervention from the participants perspectives • Increase capacity of groups to take action to improve their own mental health	• MSC stories • Meeting summary report
**9**	**Mental health and HIV** • Conducting FGD 2: links between mental health and HIV • Feedback on MSC story selection process	• Learn about participants perspectives on links between mental health and HIV • Develop awareness of relationship between poor mental health and engagement in HIV risk behaviours • Highlight MSC stories selected as significant by participants and compare with those selected by NGOs	• FGD transcripts • Meeting summary report
**10**	**Bringing it all together** • Finalising action plans • Administering post intervention questionnaires • Celebration	• Capacity to take action plan forward • Highlight achievement of groups	• Post intervention questionnaires: -WHO QOL-BREF -GHQ12 -Health Risk Questionnaire • Meeting summary report

It is anticipated that the groups will continue to meet after the project is completed with support from the NGOs, some of which already provide services to widows of IDUs.

### Data collection

A range of quantitative and qualitative data will be collected to assist with documentation of the PAG process and assessing the impact of the process on the lives of the women. While it is important to ensure that enough data are collected to facilitate assessment of the intervention, it is also important not to over-burden the women with a demanding schedule of data collection.

#### Quantitative methods

Three brief questionnaires will be completed by the women at the first and the last PAG session to assess changes in their quality of life and engagement in HIV risk behaviours. The questionnaires are: a short version of the WHO Quality of Life instrument (WHOQOL-BREF), the General Health Questionnaire (GHQ12) [27]; and a Health Risk Questionnaire developed specifically for the study. The WHOQOL-BREF and the GHQ12 are usually self-administered but assisted administration is possible for people with low literacy. The value of using the WHOQOL-BREF and GHQ12 is that they are standardised and have been used in other states of India, enabling comparisons on measures of mental health and wellbeing. The questionnaires have been translated into local languages, back translated and piloted with literate and non-literate women. The research teams and peer facilitators assessed the sensitivity and appropriateness of all questions before they were included in the study.

#### Qualitative methods

Two focus group discussions (FGDs) will be conducted during the study. The first FGD will be conducted during the second PAG meeting and focuses on concepts and determinants of mental health for women generally and for widows of IDUs specifically, and strategies for enhancing mental health. The second FGD will be conducted in the ninth session with a focus on the links between women's mental health and HIV. Indicative themes for this FGD include the impact of HIV on women's mental health and the relationship between mental health and engagement in HIV risk behaviours. The FGD question guides were developed in collaboration with the local research teams and informed by the literature and the purpose of the project. The transcripts from the FGDs will be translated into English by the research officers and analysed collaboratively.

The Most Significant Change (MSC) approach will be used to evaluate the impact of the intervention from the perspective of the women. MSC is a qualitative, participatory approach to monitoring and evaluation used in development projects. In this study, we will collect 'stories of change' from the PAG participants and systematically select those stories that best represent the most significant change. The reasons why particular stories are selected will be documented [28,29].

Finally the research officers, NGO liaison workers and peer facilitators will gather at the end of each meeting to document the PAG process so that it can be repeated or adapted in the future.

## Results

Pending analysis

## Discussion and conclusion

This intervention study is expected to (1) develop an understanding of the women's perspectives on mental health and well-being and the links between mental health & HIV; (2) raise awareness of the importance of mental health among a group of vulnerable women; (3) assess the capacity of the intervention to improve the women's quality of life and well-being and its potential to reduce engagement in HIV risk behaviours; (4) assess the feasibility of working with groups of vulnerable women to develop action plans for promoting mental health; (5) develop local capacity in research participation; and (6) promote better links between the IDU widows and the NGOs working in HIV prevention.

A dissemination workshop will be held at the end of the study so that the findings can be shared with funders, government agencies, NGOs and participants, in order to inform future strategies to improve the mental health and well-being of widows of IDUs and to improve their access to existing services.

Finally, we hope to contribute to the evidence in relation to the use of community based participatory interventions to promote mental health in development settings and the potential for these interventions to contribute to HIV prevention strategies.

## Abbreviations

AI – Appreciative Inquiry

AIHI – Australian International Health Institute

AIDS – Acquired Immuno Deficiency Syndrome

DFID – Department for International Development

EHA – Emmanuel Hospital Association

FGD – Focus Group Discussion

GHQ12 – General Health Questionnaire 12

HIV – Human Immuno Deficiency Virus

IDUs – Injecting Drug Use/Users

MSC – Most Significant Change

NGO – Non Governmental Organization

NGO liaison worker – Non Government Organization liaison worker

NIMHANS – National Institute of Mental Health & Neurosciences

PAG – Participatory Action Group

PAR – Participatory Action Research

WHO – World Health Organization

WHOQOL BREF – WHO Quality of Life Abbreviated tool

## Competing interests

The author(s) declare that they have no competing interests.

## Authors' contributions

HH, AD and MK were involved in conception of the study. HH, AD, MK and PC were involved in design of the study. AD and MK drafted the paper with contributions from HH and PC. All authors read and approved the final manuscript.

## Pre-publication history

The pre-publication history for this paper can be accessed here:


